# What is the value of anti-Müllerian hormone in predicting the response to ovarian stimulation with GnRH agonist and antagonist protocols?

**DOI:** 10.1186/s12958-015-0049-5

**Published:** 2015-06-10

**Authors:** Jure Knez, Borut Kovačič, Maruška Medved, Veljko Vlaisavljević

**Affiliations:** Department of Reproductive Medicine and Gynaecologic Endocrinology, University Medical Centre Maribor, Ljubljanska 5, 2000 Maribor, Slovenia

**Keywords:** Anti-Müllerian hormone, Ovarian stimulation, Excessive response, Poor response, GnRH antagonist, GnRH agonist

## Abstract

**Background:**

Anti-Müllerian hormone (AMH) is a marker of the ovarian reserve with promising prognostic potential in reproductive medicine. We aimed to evaluate the prognostic ability of AMH for predicting excessive or poor responses to ovarian stimulation using gonadotrophin-releasing hormone (GnRH) agonist and GnRH antagonist protocols in patients undergoing medically assisted reproduction (MAR) procedures.

**Methods:**

This retrospective analysis included 623 women who underwent ovarian stimulation for medically assisted reproduction. AMH level measurements were acquired from all couples within six months of the initiation of ovarian stimulation.

**Results:**

AMH was significantly correlated with the number of retrieved oocytes, and age was not relevant in a multivariate regression analysis (unstandardized regression coefficient of 1.130, 95 % confidence interval 0.977-1.283). AMH was a better predictor of both excessive (>19 oocytes) and poor (<4 oocytes) ovarian response than age (areas under the curve (AUCs) of 0.882 and 0.816, respectively). When stratified according to the stimulation protocol (a long GnRH agonist versus a GnRH antagonist protocol), AMH retained its high predictive value for excessive and poor responses in both groups. Serum AMH levels exhibited a strong correlation with the level of the response to ovarian stimulation.

**Conclusions:**

AMH is an independent and an accurate predictor of excessive and poor responses to GnRH agonist and GnRH antagonist protocols for ovarian stimulation.

## Background

The accurate prediction of the response to ovarian stimulation is a valuable diagnostic step in the process of medically assisted reproduction (MAR). Due to the current trend of delaying childbearing to a later time in the reproductive lifespan, MAR represents an increasingly important part of the diagnostics and treatment of subfertile couples. Although there is a clear relationship between declining fertility and female age, this relationship is highly variable [1, 2]. Therefore, a number of endocrine, echographic and functional ovarian reserve tests have been developed [3]. The aims of these tests are to facilitate the optimisation of therapy before initiating medically assisted reproductive treatment and to avoid potential unfavourable results [4]. A significant improvement in the safety of the patients undergoing assisted reproductive procedures has been achieved in the last decade, and an unexpected excessive response associated with a risk of ovarian hyperstimulation syndrome (OHSS) is no longer an acceptable outcome. Ovarian reserve tests should help to identify women who are prone to OHSS while simultaneously diagnosing women who are likely to respond poorly or have a low chance of treatment success. Many ovarian reserve tests, such as assessments of the basal follicle stimulating hormone levels (FSH), are part of currently used routine fertility diagnostic work-ups, although their abilities to correctly assess the ovarian reserve are very limited [3].

In the last decade, anti-Müllerian hormone (AMH) has emerged as an important marker of ovarian reserve. AMH is a homodimerous glycoprotein and a member of the transforming growth factor-β superfamily [5]. In females, it is secreted exclusively by the granulosa cells of the ovary. The function of AMH is to inhibit primordial follicle recruitment and decrease the sensitivity of preantral follicles to FSH [6, 7]. Hence, AMH plays an important role in the intrafollicular and interfollicular coordination of follicle development [8]. Due to this function, elevated AMH levels have been suggested to be responsible for the follicular arrest that has been observed in PCOS patients [9, 10]. AMH is primarily secreted by the preantral and small antral follicles of sizes up to 6–7 mm [11, 12]. In the larger follicles, the expression begins to decline until it gradually becomes undetectable in the large, dominant follicles [12, 13].

AMH is not expressed by atretic follicles or during the FSH-dependent, final stages of follicular growth. Thus, the basal levels of AMH more accurately reflect the total developing follicular cohort [14]. It has been shown that the serum levels of AMH are more accurately correlated with the number of antral follicles in the ovary than are the basal FSH levels [15], and the levels of AMH exhibit low levels of fluctuation across the menstrual cycle and across several consecutive menstrual cycles [16–18]. Therefore, AMH levels can be measured without significant bias related to the specific timing of the measurement in terms of the menstrual cycle [18].

However, the value of AMH has primarily been studied in patients undergoing ovarian stimulation using the long GnRH agonist protocol. Only a few studies have investigated the value of AMH in in GnRH antagonist cycles [19–21]. It remains to be confirmed whether AMH has a comparable ability to predict the ovarian response to the latter protocol of ovarian stimulation [22]. Our trial was designed to determine the ability of AMH to predict the ovarian response following ovarian stimulation with long GnRH agonist and GnRH antagonist protocols.

## Methods

### Study design

Patients with measured AMH levels who had undergone ovarian stimulation for IVF or intracytoplasmic sperm injection (ICSI) procedures were included in the retrospectively designed study. All patients from January 2011 to December 2014 were included. Patients were included regardless of age, and all indications for IVF or ICSI treatment were considered as inclusion criteria for the study. Couples undergoing IVF or ICSI in the natural menstrual cycle without applied exogenous ovarian stimulation were excluded from the study. AMH is measured as a part of the routine clinical practice in our unit, and ethical approval for the study was therefore not required.

### AMH measurement

Blood serum AMH assessments were performed as a part of the routine fertility diagnostics. All measurements were performed with 6 months of the initiation of ovarian stimulation. This approach has been proven to assure consistency in the predictive value of AMH [23]. Blood was drawn in serum tubes and stored at −80 °C until the analysis. All samples were analysed using an AMH Gen II ELISA kit (Beckman-Coulter, Webster, USA). The analytical sensitivity of the assay was 0.08 ng/mL, and the intra-assay and inter-assay coefficients of variation were than 5.4 % and 5.6 %, respectively. All values are presented in ng/mL. The conversion factor from pmol/L to ng/mL is 7.143.

### Ovarian stimulation

The patients were treated with either a long GnRH agonist (triptorelin, Diphereline; Ipsen, France) or a GnRH antagonist (cetrorelix, Cetrotide; Merck Serono, Switzerland) protocol, and these protocols have been described in detail in our previous publications [24]. The ovarian stimulation protocol was chosen after discussing the risks and benefits of each approach with the patient. Patients in their first ovarian stimulation cycle and those considered to be at high risk for OHSS were advised to utilise the GnRH antagonist protocol.

Briefly, all cycles were synchronised using oral contraceptive (OC) pre-treatment. The time of OC usage could vary (minimum of 18 and maximum of 35 days) to synchronise the menstrual cycles of the patients in the group. In the case of the long GnRH agonist protocol, seven days before the last OC pill was taken, the patients began with the administration of 0.1 mg triptorelin. In the case of the antagonist protocol, ovarian stimulation was initiated 2 days after the last pill was taken, and 0.25 mg of cetrorelix was started on a fixed protocol beginning on day 6 of the stimulation. Ovarian stimulation was initiated by the administration of a starting dose of 150–300 I.U. of recombinant FSH (Gonal-F, Merck Serono, Switzerland) or highly purified HMG (Menopur, Ferring, Switzerland). The dose could be adjusted on day 6 of the stimulation according to the level of the ovarian response as demonstrated by ultrasound. The final oocyte maturation was accomplished with 6500 I.U. of human chorionic gonadotrophin (hCG) when three leading follicles of 17 mm were observed on ultrasound. The oocyte retrieval was planned for 35 h after the hCG administration.

### Main outcomes

The primary goal of our study was to evaluate whether AMH level measured prior to MAR treatment was correlated with the level of ovarian response. Furthermore, the abilities of AMH to successfully predict excessive and poor responses were evaluated. Excessive responses were defined as more than 19 retrieved oocytes or the cancellation of the treatment cycle due to a high OHSS risk before the oocyte collection. This threshold was adopted in accordance with previously published studies because such a response is considered to indicate a high risk of OHSS for the patient [14, 25–27]. The threshold for poor response was set at <4 oocytes, which complied with the accepted Bologna criteria, or the exclusion from the stimulation due to a low response [28]. In the second part of the study, we aimed to assess whether the predictive abilities of AMH in terms of excessive and poor responses differed according to the ovarian stimulation protocol applied; i.e., the long GnRH agonist protocol or the GnRH antagonist protocol.

### Statistical analysis

The patients’ characteristics (age, number of previous MAR attempts, duration of stimulation, and total dose of gonadotrophins required) were compared with respect to the ovarian stimulation response. Analyses of variance (ANOVAs) or Kruskal-Wallis tests were used depending on the distribution of the dependent variable. Additionally, Spearman rank correlation coefficients were calculated to evaluate whether the number of retrieved oocytes was correlated with AMH and patient age. Next, the variables that were correlated with the number of retrieved oocytes (age, AMH and total dose of gonadotrophins) were used to construct a multivariate linear regression model to identify and calculate the coefficients for the factors that were independently related to the number of retrieved oocytes.

Furthermore, to assess the predictive abilities of AMH for excessive and poor responses, receiver-operating characteristic (ROC) curves for the AMH levels were constructed. The sensitivities and specificities were calculated for selected cut-off levels. To assess possible differences in the predictive ability of AMH with regard to the utilised stimulation protocol (long GnRH agonist versus GnRH antagonist protocol), the area under each ROC curve was compared using the method described by DeLong. A value of 0.05 was used as the indication of statistical significance. For the statistical analyses, the SPSS 20.0 (SPSS Inc.) and STATA 12.0 (StataCorp LP) software packages were used.

## Results

### Patients’ characteristics

Overall, 623 women who underwent IVF or ICSI procedures were included in the final analysis. The mean age of included patients was 34.8 ± 4.6, and the mean number of retrieved oocytes was 8.4 ± 6.6. Excessive responses were observed in 46 (7.4 %) women, and 162 (26.0 %) were categorised as poor responders. Table 1 shows the patients’ characteristics according to the levels of the ovarian responses. The levels of AMH and the ages of the patients were significantly different between the different ovarian response groups.

**Table 1:** Patient characteristics according to the ovarian response level

No. of retrieved oocytes	≤3	4-10	11-19	≥20	TOTAL	*p*
No. of patients	162	277	138	46	623	
Demographics						
Age (mean, SD)	36.3 (4.3)	35.0 (4.4)	33.5 (4.8)	32.3 (4.4)	34.8 (4.6)	<0.001
Previous MAR attempts (median, IQR)	0 (0–3)	1 (0–3)	0 (0–3)	0 (0–2)	0 (0–3)	0.233
AMH (median, IQR)	0.40 (0.17- 1.2)	1.35 (0.62- 2.59)	3.10 (1.87- 5.25)	5.88 (3.25- 10.60)	1.49 (0.52-3.29)	<0.001

*IQR* interquartile range

*SD* standard deviation

In 217 (34.8 %) of the patients, the long agonist protocol was applied for ovarian stimulation, and the proportions of patients who underwent the long agonist protocol were comparable across all of the ovarian response groups (Table 2). The patients were stimulated for a median of 10 days with a median total gonadotrophin dose of 2250 IU. The lo-responding patients required higher doses of gonadotrophins on average compared with the normal-responding and the high-responding patients.

**Table 2:** Ovarian stimulation parameters according to the ovarian response level

No. of retrieved oocytes	≤3	4-10	11-19	≥20	TOTAL	*p*
No. of patients	162	277	138	46	623	
Protocol used (agonist/antagonist)	47/115	102/175	53/85	15/31	217/406	0.237
Duration of stimulation (median, IQR)	10 (9–12)	10 (9–12)	10 (9–11)	10 (9–11)	10 (9–12)	0.603
Total gonadotrophin dose I.U. (median, IQR)	2625 (2100–3300)	2250 (1725–2850)	1800 (1425–2400)	1500 (1350–1800)	2250 (1650–2850)	<0.001
Outcome						
No. of oocytes (median, IQR)	2 (1–3)	6 (5–8)	14 (12–16)	23 (21–26)	6 (3–12)	<0.001

*IQR* interquartile range

### AMH and age in relation to ovarian response

The levels of AMH exhibited a strong positive correlation with the number of retrieved oocytes according to a Spearman’s rank correlation (R = 0.667, *p* < 0.001). In contrast, age exhibited a weak but statistically significant negative correlation with the number of retrieved oocytes (R = −0.272, *p* < 0.001). After the construction of a multivariable linear regression model, only AMH and not patient age was significantly and independently correlated with the number of retrieved oocytes (unstandardized coefficient and corresponding 95 % confidence interval of 1.130 and 0.977-1.283, respectively, Table 3).

**Table 3:** Linear regression coefficients (95 % confidence intervals) for the changes in the number of retrieved oocytes

Variable	Unadjusted linear regression coefficient (95 % CI)	*P*
AMH	1.130 (0.977 to 1.283)	<0.001
Age	−0.075 (−0.169 to 0.018)	0.114
Total dose of gonadotrophins	−0.001 (−0.002 to −0.001)	<0.001

### AMH and its predictive ability for the ovarian response

**Fig. 1:**
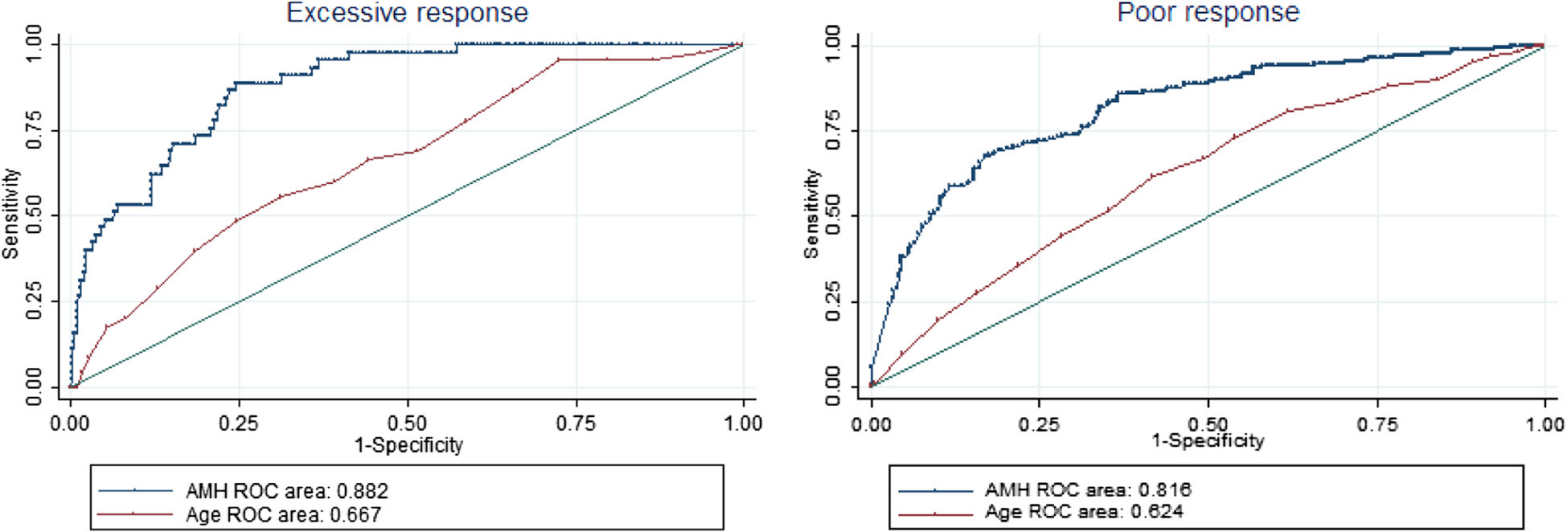
Receiver-operating characteristic curves for age and Anti-Müllerian hormone for the prediction of excessive (≥20 oocytes) and poor (≤3 oocytes) responses. (AMH: Anti-Müllerian hormone; ROC area: area under the receiver-operating characteristic curve)

In the next step of the study, the abilities of AMH to predict excessive and poor responses were analysed. The predictive abilities of AMH and age are presented in Fig. 1. AMH performed significantly better than age in terms of predicting excessive responses; the areas under the curve (AUCs) and the corresponding 95 % confidence intervals (CIs) were 0.882 (0.840–0.924) and 0.667 (0.587–0.747), respectively (*p* < 0.001). A similar pattern was observed for the poor responses; the AUC (95 % CI) for AMH was 0.816 (0.777–0.855), and that of age was 0.624 (0.575-0.673; *p* < 0.001). Furthermore, sensitivity analyses were performed for different AMH cut-off levels to improve the predictions of excessive and low responses. The best threshold for predicting an excessive response was found to be 3.07 ng/mL with a sensitivity of 83.0 % and a specificity of 78.0 %, which corresponded to positive and negative likelihood ratios (LRs) of 3.8 and 0.2, respectively, and a positive predictive value (PPV) and negative predictive value (NPV) of 23.1 % and 98.3 %, respectively. For the prediction of poor response, the threshold was set at 0.66 ng/mL, which resulted in a sensitivity of 83.7 %, a specificity of 66.7 %, a positive LR of 2.49, a negative LR of 0.2, a PPV of 46.9 %, and a NPV of 92.1 %.

**Fig. 2:**
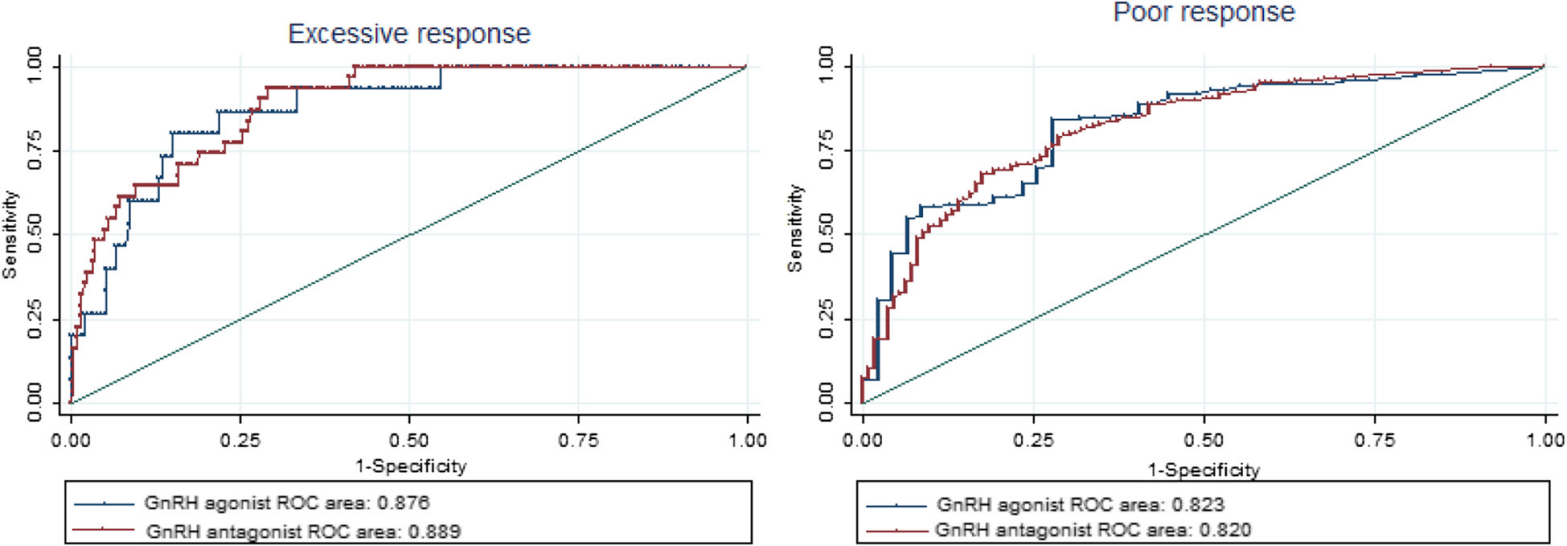
Receiver-operating characteristic curves for the prediction of excessive (≥20 oocytes) and poor (≤3 oocytes) responses according to the applied stimulation protocol. (ROC area: area under the receiver-operating characteristic curve)

Finally, we aimed to investigate whether the predictive ability of AMH was affected by the ovarian stimulation protocol employed in the treatment. Hence, ROC curves were constructed for excessive and poor response prediction according to the applied long GnRH agonist or GnRH antagonist protocol (Fig. 2). These curves revealed that the predictive value of AMH for excessive responses was unaltered by the protocol of ovarian stimulation [p = 0.79; AUCs (95 % CI): 0.876 (0.797-0.956) vs. 0.889 (0.841-0.938) for the GnRH agonist and antagonist protocols, respectively]. Similarly, the predictive values for poor responses were also comparable in both protocols of stimulation [p = 0.94; AUC: 0.823 (0.756-0.890) vs. 0.820 (0.775-0.866), respectively].

## Discussion

In the present study, we demonstrated a robust correlation of AMH with the level of ovarian stimulation response and proved the value of AMH as an independent and accurate predictor of the excessive and poor responses. Since the first clinical papers about AMH were published a decade ago, the use of AMH in reproductive medicine has become widespread [29] because many reports of the hormone’s unique characteristics have indicated that it is useful as a reliable marker of the ovarian reserve [3, 5, 13, 15, 26, 29]. Given these findings, when initiating ovarian stimulation for assisted reproduction, the ‘one size fits all’ approach is certainly no longer appropriate in contemporary clinical practice [30]. The application of reliable ovarian response predictors is becoming increasingly important. Our study confirmed only a weak correlation of age with the number of retrieved oocytes, which is consistent with the results of previously published trials [31]. AMH is a much more reliable and independent marker of ovarian response and should thus be considered when initiating ovarian stimulation treatment. The gonadotrophin dose and possible protocol modifications should be tailored to each individual, and AMH could be a useful component in these algorithms [22, 32].

Our data revealed high predictive values of AMH for excessive and poor responses, and such predictive values are prerequisites of a reliable marker. However, choosing an appropriate ‘cut-off’ level requires the assessment of the eventual benefits versus the harms of the possible misclassification of patients. Regarding excessive responses, the threshold of 3.07 ng/mL was shown to result in a sensitivity of 83.0 % and a specificity of 78.0 %. Patients with AMH levels above this threshold should be considered to be at high risk of developing OHSS, and more intense monitoring of ovarian stimulation is warranted. Moreover, the dose of gonadotrophins should be individualised regardless of the patients’ age prior to initiating the first ovarian stimulation cycle. Such optimisation also includes opting for the GnRH antagonist stimulation protocol rather than the long GnRH agonist protocol, which is by itself is related to a lower incidence of OHSS [33]. Coupled with the possibility of replacing hCG with a GnRH agonist for final oocyte maturation triggering and the additional possibility of freezing all of the developed embryos, this procedure allows for nearly complete avoidance of the threat of threatening OHSS [34, 35].

On the opposite end of the spectrum of ovarian stimulation outcomes, we have shown that at the threshold level of 0.66 ng/mL, AMH can serve as a good predictor of poor responses. However, this finding should be interpreted with caution. The threshold for a poor response should not be used as the criterion for denying treatment to a patient [36] because a substantial portion of patients below this threshold level are likely to respond well to ovarian stimulation. A false positive test might deter these patients from successful treatment. Hence, the abnormal ovarian reserve test should only be used as a tool to assist the clinician in counselling the patients about their potential for success and choice of the optimal treatment plan.

Comparing the cut-off levels determined by our study to those of previous reports revealed significant variation in the thresholds that have been determined [14, 25, 26, 31, 37–40]. This variation can largely be attributed to the lack of standard ‘poor response’ and ‘excessive response’ definitions. However, when assessing these numbers, the method of AMH detection must also be taken into account. In our trial, we used the Beckman-Coulter Gen II AMH enzyme immunoassay, and this method should be used to provide future standardisation of AMH measurements [41]. Specifically, the source of the significant differences between the results could be the methodology of the measurements because studies have shown that AMH levels measured with the Diagnostic System Laboratories (DSL) assay are ~30 % lower than those measured with the Gen I immunoassay [41, 42]. However, the Gen II immunoassay was calibrated to levels previously obtained with the Gen I AMH immunoassay [41].

Currently, the available data regarding the value AMH as a prognostic marker of the ovarian response in GnRH antagonist stimulation cycles remains limited [21, 22]. Considering our data, AMH can be used to predict excessive and poor responses to GnRH agonist and GnRH antagonist ovarian stimulation cycles with the same level of confidence. However, our study is limited by its retrospective design, and these findings should be further confirmed in future, prospectively designed studies.

## Conclusions

In conclusion, our data demonstrate the unique prognostic ability of AMH to predict the response to ovarian stimulation. Because such predictions are of paramount importance when counselling patients who are undergoing assisted reproductive procedures, AMH levels should be determined before embarking on infertility treatments.
